# miR-27 and miR-125 Distinctly Regulate Muscle-Enriched Transcription Factors in Cardiac and Skeletal Myocytes

**DOI:** 10.1155/2015/391306

**Published:** 2015-06-28

**Authors:** Estefania Lozano-Velasco, Jennifer Galiano-Torres, Alvaro Jodar-Garcia, Amelia E. Aranega, Diego Franco

**Affiliations:** Cardiovascular Research Group, Department of Experimental Biology, University of Jaén, 23071 Jaen, Spain

## Abstract

MicroRNAs are noncoding RNAs of approximately 22–24 nucleotides which are capable of interacting with the 3′ untranslated region of coding RNAs (mRNAs), leading to mRNA degradation and/or protein translation blockage. In recent years, differential microRNA expression in distinct cardiac development and disease contexts has been widely reported, yet the role of individual microRNAs in these settings remains largely unknown. We provide herein evidence of the role of miR-27 and miR-125 regulating distinct muscle-enriched transcription factors. Overexpression of miR-27 leads to impair expression of *Mstn* and *Myocd* in HL1 atrial cardiomyocytes but not in Sol8 skeletal muscle myoblasts, while overexpression of miR-125 resulted in selective upregulation of *Mef2d* in HL1 atrial cardiomyocytes and downregulation in Sol8 cells. Taken together our data demonstrate that a single microRNA, that is, miR-27 or miR-125, can selectively upregulate and downregulate discrete number of target mRNAs in a cell-type specific manner.

## 1. Introduction

MicroRNAs are noncoding RNAs of approximately 22–24 nucleotides which are capable of interacting with the 3′ untranslated region of coding RNAs (mRNAs), leading to mRNA degradation and/or protein translation blockage [1]. Understanding microRNA biogenesis has been greatly achieved; however, knowledge about the tissue distribution and functional consequences remains more elusive. In recent years, an increasing body of evidence has demonstrated a highly relevant role of microRNAs in multiple aspects of cardiac development and diseases [2, 3].

Functional evidence of the role of microRNAs in developing heart was demonstrated by selective inhibition of* Dicer* in tissue-restricted manner. Conditional ablation of* Dicer* using Nkx2.5Cre driver mice resulted in embryonic lethality with pericardial oedema and cardiac hypoplasia [4, 5]. Furthermore,* Dicer* inhibition using alpha-MHC-Cre mice also resulted in cardiac developmental impairment [6]. Thus, these studies highlight the importance of microRNA biogenesis for heart development. In addition, diverse studies have provided evidences of differential expression of microRNAs during heart development, both during embryogenesis [7–9] and at postnatal stages [10, 11] supporting a pivotal role of microRNAs during heart development. Moreover, recent studies reported microarray analyses which determine whether miRNAs are deregulated in common cardiovascular physiopathological conditions, such as hypertrophic and/or dilated cardiomyopathy, heart failure, or atrial fibrillation [12–17]. Taken together these data demonstrate a key role for microRNAs in cardiac development and disease. However, the role of individual microRNAs in these settings remains largely unknown.

We have previously reported a discrete number of differentially expressed microRNAs during cardiac development and we further elaborated on the functional role of miR-27 as regulator of the transcription factor* Mef2c* [7]. Furthermore, we recently reported that a large number of these microRNAs also display differential expression during iPS-derived cardiomyogenesis [18]. Among those, miR-125 displayed increased expression during both cardiac development and iPS-derived cardiomyogenesis, suggesting that it might play a pivotal role during muscle development. We therefore went on into this study dissecting the discrete role of miR-27 and miR-125, respectively, regulating muscle-enriched transcription factors.

## 2. Material and Methods

### 2.1. Cell Culture and MicroRNA Transfection Assays

HL1 cells (6∗10^5^ cells per well) [19] and Sol8 (ATCC, USA) cells were transfected with corresponding pre-miR (Ambion, USA), respectively, at 50 nM using lipofectamine 2000 (Invitrogen, USA) according to manufacturer's guidelines. Negative controls included nontransfected cells as well as FAM-labeled pre-miR negative control transfected cells, which also allowed transfection efficiency evaluation. In all cases, transfection efficiencies were greater than 50%, as revealed by observation of FAM-labeled pre-miR transfection. After 4 hours after transfection, HL1 cells were cultured in appropriate cell culture media and collected after 48 hours as previously reported [7, 20].

### 2.2. qRT-PCR Analyses

mRNA qRT-PCR was performed in Mx3005Tm QPCR System with an MxPro QPCR Software 3.00 (Stratagene, USA) and SSOFast EvaGreen detection system (BioRad, USA). Two internal controls, mouse*β-actin* and* Gapdh*, were used in parallel for each run. Each PCR reaction was performed at least three times to obtain representative averages. Primers sequences are provided in Table 1.

MicroRNA qRT-PCR was performed using Exiqon LNA microRNA qRT-PCR primers and detection kit according to manufacturer's guidelines. All reactions were always run in triplicate using 5S as normalizing control, as recommended by the manufacturer. The Livak & Schmittgen method was used to analyze the relative quantification RT-PCR data [21] and normalized in all cases taking as 100% the wild-type (control) value, as previously described [22].

## 3. Results

### 3.1. Search for Muscle-Enriched Transcription Factor Candidate Targets for miR-27 and miR-125

Using TargetScan search engine, mouse miR-27 is predicted to target over a thousand genes. Hand-curate literature search on PubMed indicating previous involvement of these predicted targets in the cardiovascular and/or skeletal muscle biology setting suggests that approximately only a third of these genes (324/1002; ~32%) might be targeted in this context. A subclassification of these genes demonstrates that approximately ~13% corresponded to transcription factors. Among them,* Runx1* and* Mef2c* have been already validated as direct miR-27 targets [7, 23]. We focus our attention on those transcription factors playing a role in either cardiac or skeletal muscle development, such as myostatin (*Mstn*), myocardin (*Myocd*), and MyoD family inhibitor (*Mdfi*). Using a similar approach, we also search for putative muscle-related transcription factors that might be putatively targeted by miR-125, which resulted in the identification of myocyte enhancer factor 2D (*Mef2d*).

### 3.2. Divergent Tissue-Specific miRNA Effects in Muscle Cells

**Figure 1:**
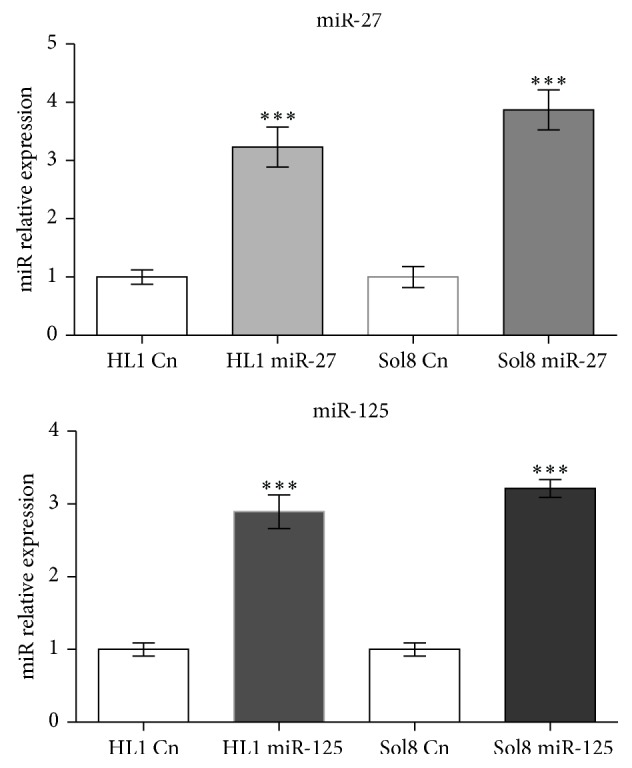
qPCR analyses of miR-27 and miR-125 expression levels in HL1 and Sol8 cells transfected with pre-miR-27 and pre-miR-125, respectively, as compared to nontransfected (lipofectamine only) control cells. Observe that a similar overexpression level is achieved for both miR-27 and miR-125 in HL1 and Sol8 cells, respectively (*n* = 3). ^***^ *P* < 0.001, ^****^ *P* < 0.0001.

**Figure 2:**
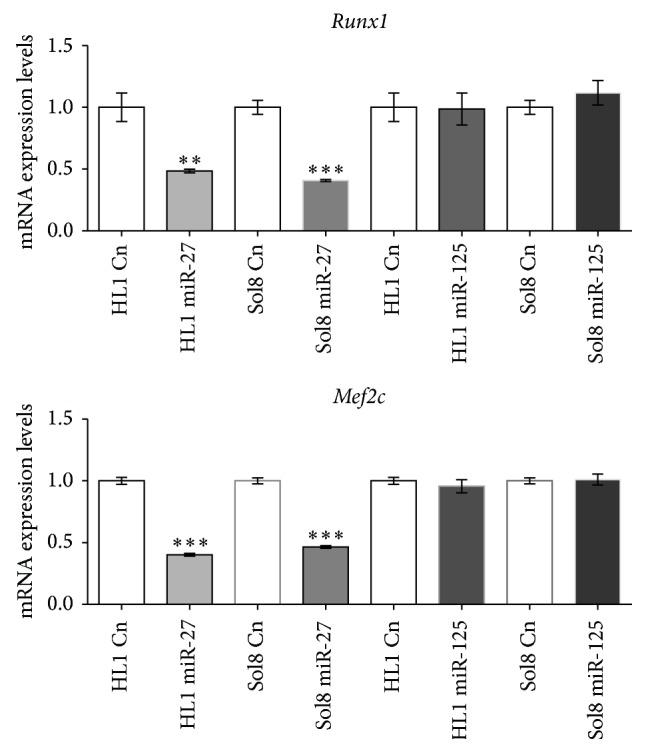
qPCR analyses of* Runx1* and* Mef2c* expression levels in HL1 and Sol8 cells transfected with pre-miR-27 and pre-miR-125, respectively, as compared to nontransfected (lipofectamine only) control cells. Note that* Runx1* and* Mef2c* expression levels are significantly downregulated in miR-27 but not in miR-125, overexpressing cells (HL1 and Sol8) (*n* = 3). ^**^ *P* < 0.01, ^***^ *P* < 0.001.

**Figure 3:**
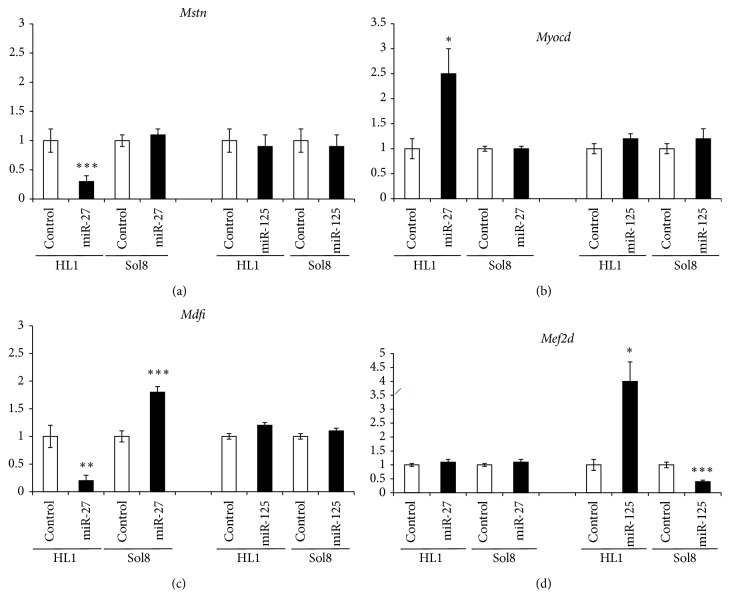
qPCR analyses of* Mstn* (a),* Myocd* (b),* Mdfi* (c), and* Mef2d* (d) expression in HL1 and Sol8 cells, respectively, transfected with miR-27 and miR-125, as stated in the corresponding panel. Observe that overexpression of miR-27 leads to downregulation of* Mstn* and upregulation of* Myocd* in HL1 cells, but not in Sol8 cells, while miR-125 overexpression does not alter any of these genes. Importantly, miR-27 overexpression downregulates* Mdfi* in HL1 cells, while it is upregulated in Sol8 cells. In the case of miR-125 overexpression, a similar effect is observed for* Mef2d* but in reverse mode; that is, miR-125 upregulates* Mef2d* in HL1 cells and downregulates it in Sol8 cells (*n* = 3). ^*^ *P* < 0.05, ^**^ *P* < 0.01, and ^***^ *P* < 0.001.

In order to dissect the functional role of miR-27 and miR-125 in muscle cells, we overexpressed these microRNAs in two distinct muscle cell types, Sol8 skeletal muscle myoblasts and HL1 atrial cardiomyocytes, respectively. After 48 hours of transfection, expression levels of these microRNAs and distinct muscle-enriched transcription factors were measured by qPCR as compared to lipofectamine nontransfected control cells.  Figure 1 demonstrates that similar levels of microRNA overexpression were achieved for miR-27 and miR-125, respectively, in HL1 and Sol8 cells. Furthermore, functional assessment of microRNA overexpression was assayed by measuring* Mef2c* and* Runx1* expression levels, since these transcription factors were previously reported as direct targets of miR-27 [7, 23].  Figure 2 shows that overexpression of miR-27 selectively results in downregulation of both* Mef2c* and* Runx1* in cardiomyocytes (HL1) and skeletal myoblasts (Sol8), whereas no significant changes were observed upon miR-125 overexpression. Interestingly, selectively overexpression of miR-27 leads to downregulation of* Mstn* in HL1 atrial cardiomyocytes but not in Sol8 skeletal muscle myoblasts, as illustrated in Figure 3. On the other hand, miR-27 overexpression leads to significant upregulation of* Myocd* in HL1 atrial cardiomyocytes, whereas no changes are observed in Sol8 cells (Figure 3). Surprisingly, overexpression of miR-27 results in downregulation of* Mdfi* in HL1 cardiomyocytes and* Mdfi* upregulation in Sol8 cells. Importantly, expression of* Mstn*,* Myocd,* or* Mdfi* is not altered in Sol8 or HL1 cells after miR-125 expression, supporting the miR-27 specificity of these effects (Figure 3).

In line with the data obtained for miR-27, overexpression of miR-125 resulted in selective upregulation of* Mef2d* in HL1 atrial cardiomyocytes and* Mef2d* downregulation in Sol8 cells. Importantly, miR-27 overexpression led to no significant changes of* Mef2d* expression neither in Sol8 nor in HL1 cells.

## 4. Discussion

MicroRNAs have been demonstrated to play essential roles in multiple biological processes, such as embryonic development, cell tissue specification, and cell proliferation, as well as in distinct pathological conditions, such as cancer and cardiovascular diseases. miR-27 has been indeed implicated in several of these contexts, such as embryonic development [7], angiogenesis [24, 25], adipogenesis [26–28], and atherosclerosis [29]. In particular, miR-27 has been reported to selectively regulate* Pax3* [20, 30],* Runx1* [23], and* Mef2c* [7]. Similarly, miR-125 has been documented to play essential roles in stem cell differentiation [31, 32] and distinct cancer types [33–35], yet its role in muscle biology is more elusive [36].

In this study, we report that miR-27 overexpression leads to selective downregulation of* Mstn* and* Mdfi* in HL1 atrial cardiomyocytes, suggesting a direct role of miR-27 regulating these genes. Surprisingly, miR-27 overexpression leads to upregulation of* Myocd* in HL1 atrial cardiomyocytes. Selective microRNA-mediated downregulation of target genes is widely documented [37, 38], although some reports also demonstrate upregulation of target genes [39, 40] such as for miR-373 [Place et al., 2007]. Thus, our data suggest that miR-27 can equally act upregulating or downregulating genes in the cardiac muscle context. Intriguingly, in Sol8 cells, miR-27 overexpression does not alter* Mstn* or* Myocd*, yet it upregulates* Mdfi*. These data suggest that miR-27 does not regulate* Mstn* and* Myocd* in the skeletal muscle context, while it upregulates* Mdfi*. Taken together these data demonstrate a cell-type specific role of miR-27 for the same target genes. A similar finding is also documented for miR-125, since miR-125 overexpression in HL1 atrial cardiomyocytes upregulates* Mef2d*, while it is downregulated in Sol8 cells.

To our knowledge this is the first report that demonstrates distinct effects for a single microRNA for the same target gene in distinct cellular contexts. In a mechanistic way, this implies that miR-27 is capable of interacting with* Mstn* and* Mdfi* 3′UTR in cardiomyocytes but not in skeletal myoblasts, while miR-125 upregulates and downregulates* Mef2d* depending on the cell context. Importantly, overexpression of miR-125 does not modify expression of* Mstn*,* Myocd*, or* Mdfi* in any cell context, while miR-27 overexpression does not alter* Mef2d* expression in HL1 or Sol8 cells. These findings reinforce the notion of a specific regulatory role of miR-27 in* Mstn*,* Myocd*, or* Mdfi* and of miR-125 in* Mef2d*, in line with TargetScan predictions. Furthermore, they suggest that either a selective blocking mechanism is operative in one cell type, for example, skeletal myoblasts, or a coadjuvant facilitating interactive factor is exclusively expressed in the other cell type, for example, cardiomyocytes. Further research is required to sort out these hypotheses. We are aware that our biological assay does not provide direct biochemical evidence of microRNA-mRNA interaction, yet it reveals the overall biological output of miR-27/miR-125 overexpression, respectively. However, it is important to realize that 3′UTR luciferase report assays in heterologous systems, such as 3T3 fibroblasts or HeLa cells, will be rather inappropriate to give the cell-type specific effects revealed in our assays.

In summary, we provide evidence that miR-27 and miR-125, respectively, can selectively upregulate and downregulate discrete number of target mRNAs in a cell-type specific manner.

## Figures and Tables

**Table 1:** List of the oligonucleotides sequences used in the qPCR assays. Note that all primers were designed using the Primer3 (http://biotools.umassmed.edu/bioapps/primer3_www.cgi) online tool, fixing the primer length to 100–200 nucleotides and an annealing temperature of 60°C. MgCl_2_ concentration was always the same since SSOFast EvaGreen Master mix was used in all qPCR experiments.

*Gapdh *	Fw: 5′-TCTTGCTCAGTGTCCTTGCTGG-3′	180 pb
	Rv: 5′-TCCTGGTATGACAATGAATACGC-3′	

*β*-*Actin *	Fw: 5′-CCAGAGGCATACAGGGAC-3′	144 pb
	Rv: 5′-TGAGGAGCACCCTGTGCT-3′	

*Myocd *	Fw: 5′-TTTTCAATTCCATCCCCAAC-3′	210 pb
	Rv: 5′-CCCAGGGATCTTTGGAATTT-3′	

*Mdfi *	Fw: 5′-CAGGCTCTGAACAGCATTGA-3′	125 pb
	Rv: 5′-GGTTCTGAGAGGTGGTCGTG-3′	

*Mstn *	Fw: 5′-GGCTCTTTGGAAGATGACGA-3′	188 pb
	Rv: 5′-GGAGTCTTGACGGGTCTGAG-3′	

*Runx1 *	Fw: 5′-TACCTGGGATCCATCACCTC-3′	164 pb
	Rv: 5′-GACGGCAGAGTAGGGAACTG-3′	

*Mef2c *	Fw: 5′-GGGGTGAGTGCATAAGAGGAC-3′	288 pb
	Rv: 5′-AGAAGAAACACGGGGACTATGGG-3′	

*Mef2d *	Fw: 5′-TCTCCCAGTCTACCCACTCG-3′	162 pb
	Rv: 5′-CAGGTGAACTGAAGGCTGGT-3′	
